# Poor Spontaneous and Oxytocin-Stimulated Contractility in Human Myometrium from Postdates Pregnancies

**DOI:** 10.1371/journal.pone.0036787

**Published:** 2012-05-10

**Authors:** Sarah Arrowsmith, Siobhan Quenby, Andrew Weeks, Theodor Burdyga, Susan Wray

**Affiliations:** 1 Department of Cellular and Molecular Physiology, Institute of Translational Medicine, University of Liverpool, Liverpool, United Kingdom; 2 Clinical Sciences Research Institute, University of Warwick, Warwick, United Kingdom; 3 Department of Women and Children's Health, Institute of Translational Medicine, University of Liverpool, Liverpool, United Kingdom; Fudan University, China

## Abstract

Prolongation of pregnancy i.e. going more than 10 days over the estimated due date, complicates up to 10% of all pregnancies and is associated with increased risk to both mother and fetus. Despite the obvious need for contractions of the uterus to end pregnancy, there have been no studies directly examining the role of uterine smooth muscle, myometrium, in the aetiology of prolonged pregnancy. This study tested the hypothesis that the intrinsic contractile characteristics of myometrium taken from women with prolonged pregnancy (>41 weeks and 3 days) was reduced compared to those delivering at term (39–41 weeks). We recruited women undergoing Caesarean Section (CS) delivery either pre-labour (n = 27) or in labour (n = 66) at term or postdates. The contractile ability of the postdates myometrium, whether spontaneous or elicited by oxytocin or high-K solution, was significantly reduced compared to term myometrium. These differences remained when adjusted for parity and other maternal characteristics. The findings remained significant when expressed per cross sectional area. Histological examination revealed no differences between the two groups. The contractile differences were however related to intracellular Ca transients suggesting an effect of [Ca] on reduced force production in the postdates group. In summary, myometrium from prolonged pregnancies contracts poorly *in vitro* even when stimulated with oxytocin and in active labour. Responses to high K^+^ and measurements of Ca suggest that alterations in excitation contraction coupling, rather than any histological changes of the myometrium, may underlie the differences between term and postdates myometrium. We show that postdates pregnancy is associated with poor myometrial activity and suggest that this may contribute to increased myometrial quiescence and hence, prolonged gestation.

## Introduction

The timely onset of labour has important implications for pregnancy outcome. Prolongation of pregnancy complicates up to 10% of all pregnancies and carries increased risk to mother and fetus [1], [2]. Despite this, little is understood about the etiology of prolonged pregnancy. With routine use of early ultrasound, dating errors as a “cause” of prolonged gestation has been greatly reduced [3], [4]. Other associations with prolonged gestation are; fetal abnormality (e.g. anencephaly), placental sulfatase deficiency, nulliparity, and prior post-term pregnancy [5], [6], [7]. It has also been suggested that genetic factors [8], male fetal gender [9] and a high maternal body mass index [10], [11], [12], [13] contribute to an increased risk of prolonged gestation. Surprisingly however, the role of the myometrium in prolonged pregnancy remains poorly investigated.

The precise sequence of events preceding human labour onset are unknown but, as suggested by Challis and Lye [14] up-regulation of a series of genes encoding contraction-associated proteins (CAPs), which includes the oxytocin receptor [15] and the gap junction protein connexin-43 [16] and changes of excitability (ion channel changes) [17], [18] and intracellular Ca homeostasis [19], [20], are considered important. For uterine contractions, Ca entry is pivotal and is likely to be implicated in mechanisms affecting uterine activation and onset of labour [21], [22]. If uterine quiescence is extended we suggest it will lead to prolongation of pregnancy.

Much of our current understanding of the function of the human myometrium has arisen from *in vitro* studies examining the contractile abilities of myometrium obtained during pre-labour elective Caesarean section (CS) at term [23]. Surprisingly, very little is known about the functional abilities of myometrium beyond term, or why some women progress beyond their ‘due date’. Clinical markers of poor uterine activity include higher rates of CS and instrumental delivery, increased labour augmentation and longer lengths of labour and postpartum haemorrhage. Notably, these markers have all been associated with post-term pregnancies [1], [24], [25], [26]. Furthermore reduced uterine activity was found using *in vivo* tocodynamometer recordings of amniotic pressure changes [27], which also suggests poorer contractility. We therefore hypothesized that the intrinsic contractile characteristics of myometrium taken from women with prolonged pregnancy would be reduced compared to those delivering at term and that these differences would persist even when labour was initiated. To the best of our knowledge, there have been no *in vitro* studies undertaken to explore the functional abilities of postdates myometrium. The present study was therefore undertaken to 1) compare the inherent spontaneous contractile properties of myometrium from term and prolonged (postdates) pregnancies, including initiation of activity, 2) determine if any contractility differences persist after labour has commenced, 3) examine contraction augmentation by oxytocin, 4) investigate if changes in intracellular Ca transients are apparent between the different tissue types, and 5) analyze the histology of postdates myometrium.

**Table 1:** Maternal demographics of women delivering by CS *not in labour* according to gestational age group.

	Term (n = 14)	Postdates (n = 9)	*P* value
**Gestational age (days)**, median (IQR)	275 (274–276)	290 (290–292)	**<0.001** *
**Maternal age (years)**, mean (SD)	31.7 (6.1)	32.9 (5.9)	0.655
**Maternal BMI (kg/m^2^)**, mean (SD)	31.1 (6.2)	30.8 (6.0)	0.913
**Birthweight (grams)**, mean (SD)	3743 (699)	3523 (528)	0.451
**Nulliparous, n (%)**	1 (7.1)	2 (22.2)	0.679
**Reason for CS**, n (%)			0.383
Previous CS	10 (71.4)	5 (55.6)	
Breech	3 (21.4)	1 (11.1)	
Maternal reason	1 (7.1)	2 (22.2)	
Fetal reason	0 (0.0)	1 (11.1)	

Gestational age refers to the gestational age at delivery and is based upon ultrasound scan taken during pregnancy booking within the first semester of pregnancy. Maternal age is age at delivery. Maternal BMI was calculated from height and weight measurements taken at pregnancy booking.

* Denotes statistical difference by students unpaired t-test, Mann-Whitney U test or Chi Square (χ^2^) test.

**Table 2:** Maternal demographics and labour details of women delivering by CS *in labour* according to gestational age group.

	Term (n = 14)	Postdates (n = 21)	*P* value
**Gestational age (days)**, median (IQR)	281 (275–286)	292 (291–293)	**<0.001** *
**Maternal age (years)**, mean (SD)	29.9 (5.7)	27.5 (5.2)	0.211
**Maternal BMI**, mean (SD)	25.3 (4.6)	27.1 (4.4)	0.272
**Birthweight (grams)**, mean (SD)	3327 (697)	3806 (683)	0.057
**Nulliparous, n (%)**	6 (42.9)	17 (81.0)	**0.026** *
**Induction, n** (%)	6 (42.9)	15 (71.4)	0.159
**Augmentation, n** (%)	5 (35.7)	16 (76.2)	**0.033** *
**Length of labour (hrs)**, mean (SD)	9 (6.9)	11 (7.5)	0.342
**Reason for CS**, n (%)			0.067
Failure to progress	6 (42.8)	8 (38.1)	
Fetal distress	2 (14.3)	10 (47.6)	
CPD	2 (14.3)	3 (14.3)	
Previous CS	2 (14.3)	0 (0)	
Breech	2 (14.3)	0 (0)	

Gestational age refers to the gestational age at delivery and is based upon ultrasound scan taken during pregnancy booking within the first semester of pregnancy. Maternal age is age at delivery. Maternal BMI was calculated from height and weight measurements taken at pregnancy booking. Induction refers to the number of women receiving labour induction with vaginal prostaglandin with or without intravenous syntocinon. Augmentation refers to the number of women receiving intravenous syntocinon. CPD, cephalopelvic disproportion.

* Denotes statistical difference by Student's unpaired t-test, Mann-Whitney U test or Chi Square (χ^2^) test.

**Figure 1:**
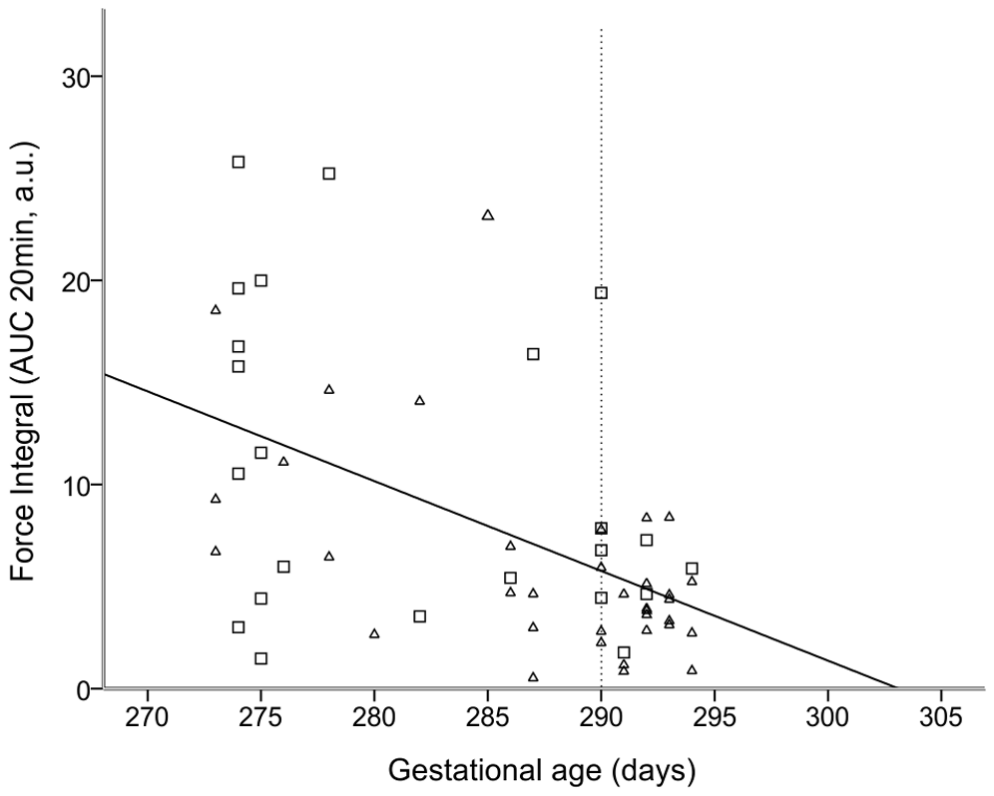
Myometrial contractility in relation to gestational age. Plot of spontaneous contractile activity (quantified as integral force of contraction/area under the contraction curve, AUC) of strips of myometrium obtained from women (n = 58) undergoing Caesarean section (CS) delivery against gestational age. Spearman's rank test found a significant negative correlation between force integral and gestational age (*r* = −0.478, *P*<0.01). Pre-labour CS women are denoted by open squares and CS in labour women are denoted by open triangles. Dotted vertical line indicates postdates delivery (≥41weeks^+3^ days).

## Methods

### Ethics Statement

This study was given a favourable ethical opinion by the North West (Liverpool East) Research Ethics Committee (REC refs 01/213, 97/190 and 09/H1002/65) and by the Research and Development Director of Liverpool Women's NHS Foundation Trust. Liverpool, UK. All women provided written informed consent for the collection of samples and subsequent analysis.

**Figure 2:**
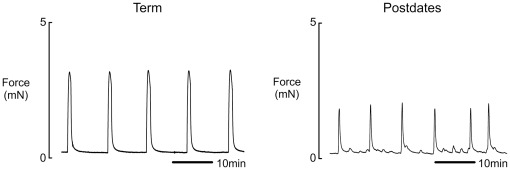
Contractile activity in term and postdates labouring myometrium. Representative isometric recordings of spontaneously contracting myometrial strips obtained from women undergoing Caesarean section in labour at term (39–41 weeks) and postdates (≥41weeks^+3^). Strips were placed under a resting tension of 2 mN and superfused continually with physiological saline solution (pH 7.4) at 37°C.

**Table 3:** Contractility parameters measured according to gestational age group.

	Gestational age group		
*All women*			
	Term (n = 28)	Postdates (n = 30)	*P* value
Force Integral (AUC, a.u.)	10.94±1.40	4.97±0.60	**<0.01** *
Force amplitude (mN)	3.60±0.49	2.07±0.37	**0.017** *
Frequency (no. 10 min^−1^)	1.14±0.12	1.87±0.28	**0.021** *
Duration (min)	1.10±0.09	0.80±0.09	**0.023** *

*Nulliparous* women			
	Term (n = 7)	Postdates (n = 19)	*P* value
Force Integral (AUC, a.u.)	12.54±2.58	4.23±0.50	**0.018** *
Force amplitude (mN)	3.49±0.61	2.34±0.56	0.266
Frequency (no. 10 min^−1^)	1.32±0.25	1.89±0.33	0.332
Duration (min)	1.35±0.21	0.65±0.06	**0.014** *

*Non-labouring* women			
	Term (n = 14)	Postdates (n = 9)	*P* value
Force Integral (AUC, a.u.)	12.86±2.21	7.05±1.66	**0.048** *
Force amplitude (mN)	4.63±0.78	1.78±0.34	**0.004** *
Frequency (no. 10 min^−1^)	1.03±0.13	2.43±0.96	0.185
Duration (min)	1.06±0.13	0.99±0.24	0.761

*Labouring* women			
	Term (n = 14)	Postdates (n = 21)	*P* value
Force Integral (AUC, a.u.)	9.02±1.74	4.05±0.50	**0.015** *
Force amplitude (mN)	2.58±0.48	2.20±0.52	0.621
Frequency (no. 10 min^−1^)	1.26±0.21	1.80±0.30	0.204
Duration (min)	1.14±0.14	0.72±0.07	**0.005** *

Contractility parameters measured are based upon a minimum period of 20 minutes stable spontaneous activity. Data is represented by mean (± S.E.M.)

* denotes significance found by Student's unpaired t-test (*P*<0.05).

### Patients and tissue preparation

We recruited 93 women undergoing CS delivery either electively (n = 27) i.e. pre-labour, or intrapartum i.e. in labour (n = 66), delivering at term or postdates at Liverpool Women's Hospital, Liverpool, UK. Gestational age at delivery was based upon ultrasound scan measurement of the crown-rump length at pregnancy booking in the first trimester. Term delivery was defined as delivery one week either side of the estimated 40 week date of delivery (39–41 weeks gestation). Prolonged pregnancy was defined as delivery on or after 290 days (≥41^+3^ weeks) gestation as the hospital protocol was to refer women to a dedicated postdates pregnancy clinic at 290 days.

**Figure 3:**
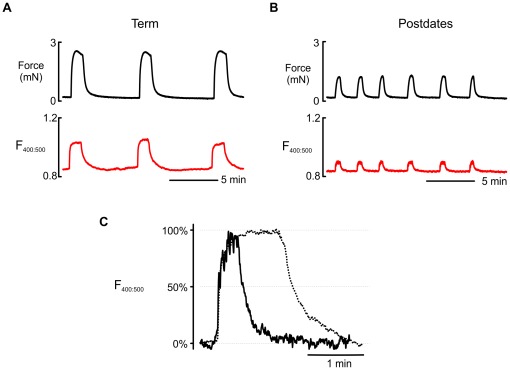
Contractile activity and Ca signalling in term and postdates non-labouring myometrium. Simultaneous recordings of spontaneous contractile activity (top, black trace) and intracellular Ca transients (Indo-1, ratio of fluorescence at 400∶500 nm, bottom, red trace) of myometrial strips obtained from women undergoing planned pre-labour CS at (**A**) term (39–41 weeks) and (**B**) postdates (≥41weeks+3). This example shows a term sample contracting particularly strongly to emphasize the difference in Ca signals. Inset (**C**) below on expanded time scale shows an overlay of Ca transients normalised by amplitude showing significantly reduced transient duration in postdates myometrium (solid black line) compared to term myometrium (dotted black line).

**Table 4:** Calcium transient data.

	Term (n = 14)	Postdates (n = 11)	*P* value
Amplitude (F400∶500)	0.094±0.010	0.068±0.006	**0.046** *
Duration (min)	1.210±0.142	0.622±0.143	**0.008** *
Integral (AUC in 20 minutes, a.u.)	0.500±0.060	0.251±0.025	**0.002** *

Transient amplitude reflects change in relative Indo-1 fluorescence ratio (F400∶F500) and is indicative of change in intracellular Ca concentration. Duration refers to duration of Ca transient and integral reflects area under the transient curve for 20 minutes of stable spontaneous activity. Data are represented by mean (± S.E.M.).

* denotes significance found by Student's unpaired t-test (*P*<0.05).

Indications for CS in labour were failure to progress (30), fetal distress (21), cephalopelvic disproportion (9), undiagnosed breech (3) and planned elective CS but presented in labour before booked date (3: the indication for all these planned CS was a previous CS). The mean maternal age at delivery was 28 years (±5.2 years) whilst the median gestational age was 291 days, equivalent to 41 completed weeks plus 4 days (41^+4^ weeks; IQR 282–293 days). The indications for CS in the non-labouring group were previous CS (n = 16), breech (n = 6), fetal reason (n = 1, abnormal fetal CTG) and maternal reason (n = 4; previous difficult vaginal delivery). The mean age of these women was 32 years (±5.8 years) and median gestational age was 278 days, (39^+5^ weeks; IQR 275–290 days). Women were included if they were able to give written, informed consent about the study before their CS. We excluded women with multiple pregnancy, serious medical complications (diabetes, pre-eclampsia, HIV/AIDS, hepatitis B or C), ketonuria, uterine hyperstimulation or use of medication likely to affect myometrial activity (e.g. antihypertensive medication).

**Figure 4:**
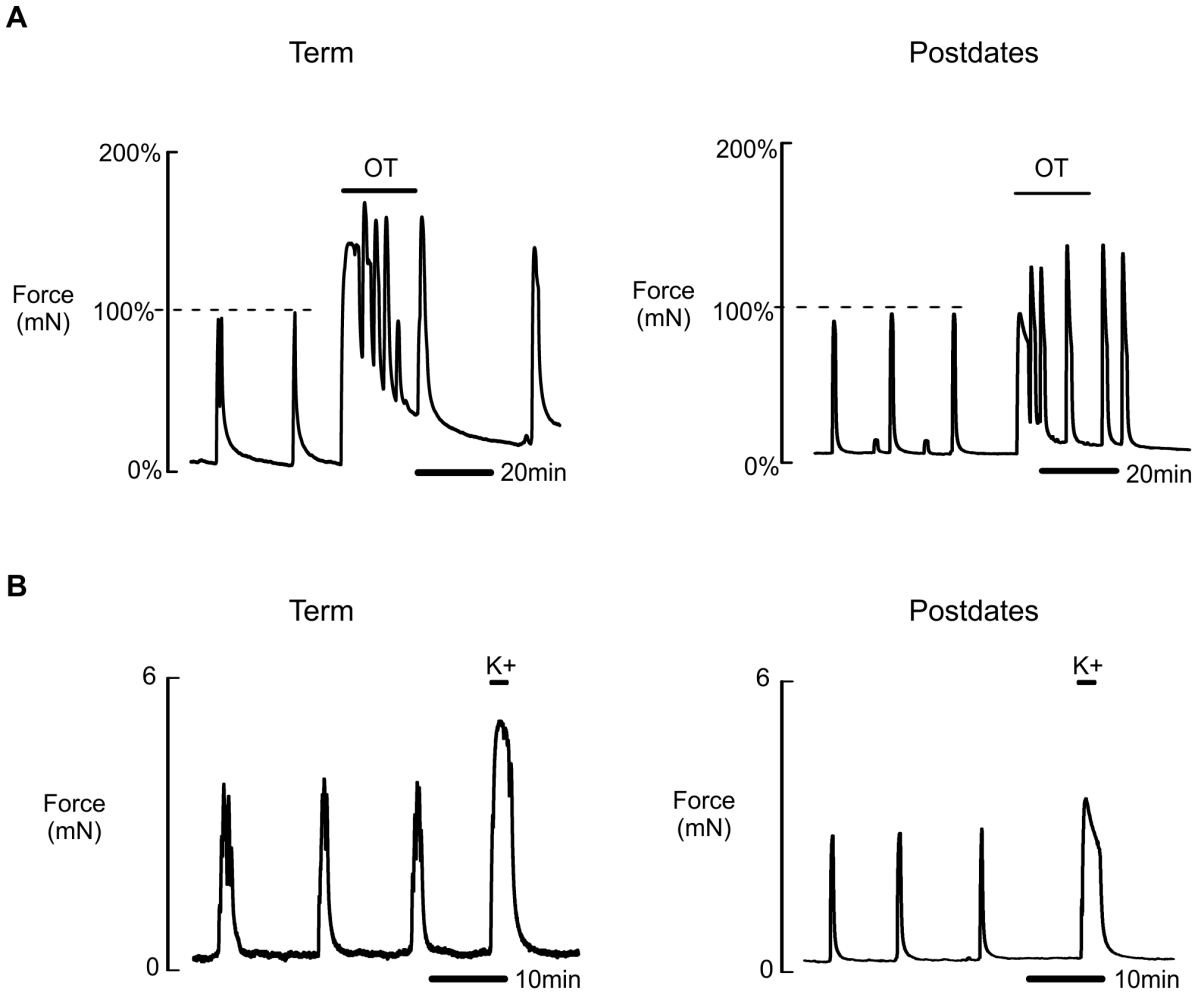
The response to oxytocin and high K^+^ in term and postdates myometrium. After establishing stable spontaneous myometrial contractions, 10 nM oxytocin (OT) (**A**) or high K^+^ (**B**) was added for 20 min and 2 min respectively. Response to OT was compared between term and postdates myometrium by measuring both the force amplitude and the percentage increase in force amplitude of contraction under OT where control activity equalled 100% (as shown in **A**). The response to high K^+^ (mean force amplitude of contraction) was compared between groups and was found to be significantly reduced in postdates myometrium compared to term.

**Table 5:** The effect of oxytocin and high K^+^ on myometrial contractility according to gestational age group.

	Gestational age group		
Oxytocin (OT, 10 nM)			
*All women*	Term (n = 28)	Postdates (n = 10)	*P* value
Force amplitude under OT (mN)	7.38±1.03	3.92±0.90	**0.022** *
% increase in force amplitude	+89.0±22.5	+91.0±14.9	0.948
Integral force under OT (AUC 20 min, a.u.)	56.5±10.7	27.3±6.7	**0.027** *
% increase in integral force under OT, (%)	+342.0±47.6	+413.3±75.2	0.437

*No therapeutic OT*	Term (n = 26)	Postdates (n = 7)	*P* value
Force amplitude under OT (mN)	7.85±1.10	3.84±1.20	**0.022** *
% increase in force amplitude	+69.3±14.2	+98.02±18.3	0.332
Integral force under OT (AUC 20 min, a.u.)	59.9±11.3	22.7±6.7	**0.008** *
% increase in integral force under OT, (%)	+345.7±49.3	290.3±49.0	0.581

High K^+^			
	Term (n = 23)	Postdates (n = 17)	*P* value
Force amplitude under high K^+^, (mN)	4.21±0.64	2.60±0.44	**0.044** *
% increase in force amplitude	+26.3±11.0	+45±14.6	0.280
Spontaneous activity (force amplitude) normalised to high K^+^ (force amplitude), (%)	85.8±3.80	73.2±6.70	0.122
Integral force under high K (AUC 2 min, a.u.)	7.2±1.07	3.9±0.60	**0.023** *

Data are represented by mean (± S.E.M.).

* denotes significance found by Student's unpaired t-test (*P*<0.05).

At operation, a full thickness biopsy (2 cm long, 1 cm wide and 1 cm thick) was obtained from the upper edge of the lower uterine segment incision. Samples were taken immediately after delivery of the baby and prior to routine administration of a bolus of oxytocin. All biopsies were immediately placed in Hanks Balanced Salt solution, stored at 4°C and used for experimentation within 12 hours of collection or histology (see below).

### Contractility and Calcium measurements

In the laboratory, biopsies were cleared of endometrium, excess blood and any fetal membranes present. Strips of myometrium 5 mm long, 2 mm wide, and 1 mm thick were cut so that the longitudinal axis was aligned with the direction of the muscle fibres. The strips were mounted, secured by aluminium clips in a 1 ml chamber bath positioned on the stage of an inverted microscope (Nikon Diaphot, WPI Ltd) and viewed with a 20× fluor objective lens. To ensure the amount of stretch applied was standardised across experiments, all strips were placed under isometric conditions with a resting tension of 2 mN. Contractility was recorded via a tension transducer (FT03, Grass Technologies, Slough, UK) attached to one end of the strip, which was connected to a data acquisition system (Axon) [28]. The strips were superfused with physiological saline (PSS; see below) at a rate of 1.5 ml/min at pH7.4 and 36°C. Under these conditions, it has been previously noted that most strips develop a steady baseline tension and achieve spontaneous contractions within 2 hours of continual superfusion [29]. Contractions stabilize with a regular and consistent pattern of activity within 60 minutes and can be recorded for many hours. Where no spontaneous activity was achieved following 2 hours of superfusion with PSS, the strip was challenged with a 2 minute application of high (40 mM) K^+^ to induce contraction and give an indication of tissue activity. If the tissue responded to high K^+^, the strip was then examined for a further 1 hour. If no further activity was recorded, the strip was changed and the protocol repeated. The same protocol was repeated for a minimum of 3 different strips from the same biopsy. Where no response to high K^+^ was achieved from a minimum of 3 different strips, the sample was classified as ‘non-contractile’. If the tissue responded to high K^+^ but did not produce spontaneous contractions, following a 3^rd^ hour of continual superfusion with PSS and despite examining 3 different strips from the same biopsy, it was classified as ‘non-spontaneous’. In some experiments, following a period of stable spontaneous activity, oxytocin (10 nM) was added to the PSS to measure the activity under uterotonic stimulation.

**Figure 5:**
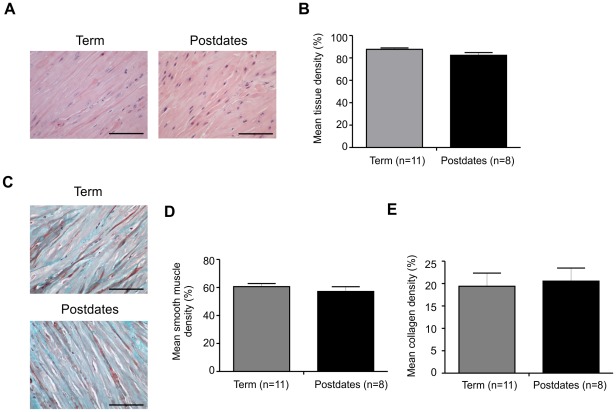
Histological examination of term and postdates myometrium. Typical examples of Haematoxylin and Eosin stained myometrial sections from term (n = 11, left) and postdates (n = 8, right) women are shown in (**A**). The percentage of field occupied by muscle cells (mean tissue density) for the two groups showed no significant difference (**B**), *P*>0.05. Sections of term and postdates myometrium stained with Masson's Trichrome are shown in (**C**, term top; postdates bottom). There was no difference in the mean densities of smooth muscle (**D**) or collagen (**E**) between gestational age groups. Scale bar represents 100 µm.

In some preparations simultaneous measurements of force and intracellular Ca were made as follows: The strips were pre-loaded for 4 hours at room temperature (21°C) in PSS containing 12.5 µM of the membrane-permeant form of the Ca-sensitive indicator Indo-1 (Indo-1 AM, Molecular probes, Invitrogen). Indo-1 was excited at a wavelength of 350 nm and light emitted at 400 and 500 nm wavelengths were recorded to measure Ca, as described in detail by us elsewhere [30]. Only preparations showing opposite changes in the F400 and F500 wavelengths were used for analysis (n = 25). Indo-1 loading as described does not affect contractile activity [31].

Following most experiments, tissue strips were weighed from which an estimation of tissue cross sectional area (cm^2^) was calculated using the following formula: W/L * D, where W = blotted wet weight (g), L = tissue length (0.5 cm standard) and D = tissue density (1.05 g/cm^3^).

### Histology

Nineteen biopsies (11 term, 8 postdates) from women delivered by CS in labour were fixed in 10% neutral-buffered formalin for 24 h, routinely processed and embedded in paraffin wax. Five-micron-thick sections were mounted onto APES (3-aminopropyltriethoxysilane) coated slides and stained with haematoxylin and eosin (H&E) or Masson's Trichrome and examined for histological differences. Sections were viewed at ×40 magnification with the observer (S.A.) blind to the origin of the sample. Images from 10 randomly selected regions per sample and per stain were captured using Eclipsenet software (Nikon, Nikon UK, Kingston upon Thames, UK). Colour-based thresholding using ImageJ freeware (NIH, Bethsheda, MD, USA) was used to measure the percentage of field (whole field = 100%) occupied by tissue, smooth muscle and collagen (H&E and Masson's Trichrome respectively). A mean average percentage tissue, percentage smooth muscle and percentage collagen per sample was obtained.

### Solutions

All chemicals were purchased from Sigma, Poole, Dorset, unless otherwise stated. Physiological saline solution (mM) was prepared as follows: 154 NaCl, 5.6 KCl, 1.2 MgSO_4_, 7.8 glucose, 10.9 HEPES and 2.0 CaCl_2_, pH 7.4.) High K^+^ (40 mM) was prepared by the isosmotic substitution of NaCl for KCl (in mM; 114 NaCl, 40 KCl). 10 nM oxytocin was prepared by addition to PSS.

### Analysis

Patient electronic clinical records were accessed and reviewed retrospectively by a senior obstetrician (SQ or AW) for details relating to the woman's labour where necessary (e.g. time in labour, dilation, reason for CS, use of therapeutic oxytocin) and demographic details (e.g. age, gestation at delivery, BMI at booking, parity etc.). Contractility measurements were made and analysed whilst blinded to patient details and labour outcomes. Labour was defined as having a record of regular contractions with associated cervical dilation greater than 3 cm. Women were then grouped according to their gestation at delivery and labouring status.

All data presented is representative of muscle strips that had been left to equilibrate for a minimum of 2 hours and developed spontaneous contractions within 3 hours of continual superfusion. Force and Ca measurements are expressed as mean ± S.E.M. with respect to a period of consistent activity, defined as a minimum of four contractions of equal amplitude and frequency. The time taken to establish consistent contractions varied (median 2 hours range, 1–3 hours). For analysis of the physiological differences between the patient groups, the different parameters measured were peak amplitude above baseline (expressed as force in mN), frequency of contractions (number of contractions in 10 minutes), duration of contraction measured at 50% of peak amplitude and contraction integral, the area under the curve (AUC) expressed in arbitrary units, au (calculated for 20 minutes of spontaneous activity or under oxytocin and for 2 minutes under high K^+^).

### Statistics

All demographic data except for gestational age, was found to be normally distributed, hence values are presented as mean ± SD and were assessed by Student's unpaired test. Gestational age is expressed as median age ± interquartile range (IQR) and was assessed by Mann Whitney U test. The Chi square test χ^2^ was used to assess categorical data which are presented as counts and percentages. For analysis of contractility and Ca data, all values represent the mean ± S.E.M. where ‘n’ is the number of samples with each representing a different woman. In some cases, results are expressed as percentage of control or high K^+^ activity where control or high K^+^ activity is equal to 100%. All data were found to be normally distributed by the Shapiro-Wilk test for normality and were compared by Student's unpaired t-test with *P*<0.05 taken as the level of significance. The association between tissue function (force integral) and gestational age was tested with Spearman's rank correlation test (correlation coefficient is provided as *r*). For histological examination of samples, a student's unpaired t-test was used to compare the mean smooth muscle and collagen densities between the two gestational age groups.

## Results

### Establishment of spontaneous contractile activity

In the laboratory we found no significant differences in the ability of term (n = 44) or postdates (n = 49) myometrium to initiate stable spontaneous contractions (63.6 and 63.2%, respectively). Nor were there any significant differences in the number of samples that were either non-spontaneously contractile (20.4 and 28.5%) or non-contractile (15.9 and 8.2%, respectively). There were also no significant differences between term and postdates myometrium, if data were analysed separately for in labour (n = 66) or not in labour (n = 27).

The demographics of the 23 women having elective CS and the 35 who had CS during labour and from whom spontaneous contractility data were obtained, are given in Tables 1 and 2 respectively. There were no significant differences in maternal age, BMI, neonatal birthweight or reason for CS between the term and postdates groups having elective CS (Table 1). There was also no difference in maternal age, BMI or neonatal birthweights between the term and postdates women having CS in labour (Table 2). Furthermore, length of labour and reason for CS did not differ significantly between the two groups but use of oxytocin augmentation was greater for postdates women, as was the number of women who were nulliparous (Table 2).

### Spontaneous uterine contractility is reduced in postdates women

Figure 1A shows the correlation between force integral and gestational age for all spontaneously active preparations (n = 58). Spearman's rank correlation analysis showed that there was a significant negative correlation between force and gestational age (*r* = −0.478, *P*<0.01).

When the data were grouped into term (n = 28) and postdate (n = 30), we found contraction amplitude, duration and AUC to be significantly reduced in postdate myometrium compared to term myometrium, although the frequency of contractions were significantly increased. These mean data are shown in Table 3, and original records in Figure 2.

### Activity normalised to cross-sectional area

To confirm that the force differences observed above were due to gestational differences and not a systematic error or bias in dissecting the myometrial strips, measurements were also made per cross sectional area (XSA, n = 42). The mean blotted wet weight of myometrial strips was 2.82 mg±0.32 (SD) and the coefficient of variation was 11.3%. There was no difference in the blotted weights of samples between gestational age groups: 2.80 mg±0.365 (SD) term samples (n = 17), 2.83 mg±0.295 (SD) postdates samples (n = 25) (*P* = 0.727). The differences in force area and amplitude remained significant when expressed per XSA (N/cm^2^): amplitude of the term group was 0.524 N cm^−2^±0.109 compared to 0.290 N cm^−2^±0.057 (*P* = 0.047) in the postdates group, and the mean AUC were 1520±304 and 765±143 au. cm^−2^ respectively (*P* = 0.017).

### Effect of parity

As shown in Table 2, a significantly greater number of postdates women in labour were nulliparous compared to the term in labour group. Thus to test whether we were detecting contractility differences in respect of gestation or parity, we compared the contractility of only nulliiparous women. The AUC remained significantly reduced in the postdates samples (n = 7 term, n = 20 postdates), as shown in Table 3, the differences in contractility were still evident. This was also the case if only the labouring group of postdate and term women were compared (n = 6 and n = 18, respectively).

### Effect of labour

To address whether entry into labour abolished the differences in contractility found in postdates women, we analysed contractility separately in samples from women labouring and not in labour. The mean data is presented in Table 3. We found that irrespective of whether labour had been initiated or not, contractility as assessed by AUC, was significantly lower in samples from postdates pregnancies. For non-labouring women, the amplitude of contraction was also significantly reduced in postdates women compared to term women not in labour, and for labouring women, duration of contraction was also significantly reduced.

Thus irrespective of labour state and parity, myometrial contractility from women with a prolonged pregnancy is reduced compared to term controls. As intracellular Ca is pivotal to contractility in myometrium, we next examined the Ca signalling in postdates myometrium.

### Changes in intracellular Calcium in postdate myometrium

Spontaneous contractile activity and intracellular Ca measurements were obtained from 25 samples; 14 term and 11 from postdates pregnancy. In all cases, the increase in [Ca]_i_ preceded the increase in tension as previously described [32]. There was a close association between Ca and force in the myometrium during spontaneous phasic contractions in samples from both gestational groups. The amplitude (relative change in ratio of fluorescence), duration and AUC of the Ca transients during 20 minutes of spontaneous activity were measured and mean values (± S.E.M.) are shown in Table 4. Typical trace recordings of spontaneous contractions and Ca transients from both gestational groups are shown in Figure 3A and B. In the postdates myometrium where contractions were smaller in force amplitude, the associated Ca transient amplitudes were also significantly smaller, compared to term myometrium, as were duration and AUC. Thus, women with postdates pregnancies have smaller Ca transients than those occurring in term myometrium, suggesting a direct effect of change in intracellular [Ca] or signalling via Ca on force production occurs in postdates myometrium.

### Effects of Oxytocin and high K^+^

We analysed contractility stimulated by the application of 10 nM oxytocin and compared it to the spontaneous contractions elicited by the same tissue strip in term (n = 28) and postdates (n = 10) myometrium. Oxytocin augmented contractile amplitude and AUC in both groups by a similar amount (Figure 4A, Table 5). Consequently, as with spontaneous activity, the mean amplitude and AUC with oxytocin elicited by postdates myometrium was significantly reduced compared to the response observed in term myometrium (Table 5). To control for any confounding effects of prior exposure to therapeutic oxytocin on the subsequent *in vitro* response to oxytocin, we also examined the data after exclusion of women receiving labour augmentation. Removal of these cases resulted in similar conclusions with both force amplitude of contraction and AUC under oxytocin being significantly decreased in the postdates myometrium compared to term myometrium (Table 5).

Similar data were obtained with high K^+^ stimulation, (n = 23, term and n = 17 postdates samples, Table 5) i.e. force was increased in both groups but the amount produced by postdates uterus was still less than high K^+^ stimulated term (Table 5 and Figure 4B). The AUC under high K^+^ was also significantly reduced in postdates compared to term myometrium. Data also show that the amplitude of spontaneous contractions when expressed as a percentage of high K^+^ (where high K^+^ amplitude = 100%) were not significantly different between the groups (Table 5).

### Histological examination of postdates compared to term myometrium

We performed light microscopic examination of haematoxylin and eosin-stained sections, for comparison of tissue density between samples and Masson's Trichrome stained sections for comparison of smooth muscle and collagen densities of myometrium from, either at term or postdate. We compared 11 contractile samples from women at term and 8 contractile samples from postdates women. We found no differences in the gross appearance of muscle cell size, organisation and separation of cells within bundles, of the myometrium from postdates samples compared to term (Figure 5A). Comparison of mean tissue density by measurement of the percent of the field of view occupied by tissue, showed no significant difference between sample groups; (Figure 5B). In sections stained with Masson's Trichrome (Figure 5C) we compared mean smooth muscle and mean collagen densities between the two gestational groups and again found no differences, Figure 5D and 5E respectively.

## Discussion

This is the first study to directly examine the *in vitro* contractility of the myometrium from women with prolonged pregnancy. We show physiological differences between contractions of term and postdates myometrium. Firstly, the myometrium from postdates pregnancies had contractions that were lower in amplitude and shorter in duration than term myometrium; thus mean AUC, which is an index of the total contractile activity, is significantly reduced compared to term myometrium. Secondly, the decreased contractility was found for labouring and non-labouring postdates myometrium. This suggests that the changes in the myometrium necessary for labour are not sufficient to overcome the underlying causes of postdates pregnancy. Thirdly, although oxytocin and high K^+^ solutions could stimulate postdate and term myometrium to a similar extent, the postdates tissue still produced significantly less force than term, as they start from lower levels of spontaneous activity. And fourthly, the data obtained cannot be explained by differences in tissue strip size or histology, as they persist when force is expressed per cross sectional area and no histological differences were found. Reductions in the Ca transients accompanying contractions were found to be associated with the reduced contractile activity in postdates samples, indicating that differences in L-type Ca channel activity or expression could underlie the reduced contractility. This reduced level of L-type Ca channel activity would also explain why both oxytocin and high K^+^ were not able to abolish the differences between term and postdates samples. Thus we suggest that poor uterine activity is an inherent property of the postdates myometrium, and that this is independent of labour onset and consistent with other clinical data, discussed later.

There are very few previous studies of post-term myometrium with which to compare our study. No other *in vitro* studies of force or Ca transients appear to have been made. Our data showing reductions in both are thus novel and provide insight in to this common complication of pregnancy. We considered that the reduced force, present even when samples were normalised to cross sectional area could be due to a decrease in muscle content in the strips from postdates women. Our histological examination however did not support this conclusion, as no differences were found in mean muscle or collagen content between the two groups. These methods were able to detect small differences in myometrium from pregnant diabetic women compared to non-diabetic women, suggesting that it is not a difficulty with sensitivity and analysis [33]. We also found no difference in the histology of postdates myometrium when we analyzed for length of labour or reason for CS (not shown). The only other histology on postdates pregnancy appears to be that of Kuc et al. [34] who reported diminished glycogen and alkaline phosphatase in myometrium from prolonged pregnancies. How these changes relate to prolonged pregnancy in a causal way is not clear. We found no differences in these tissues initiating spontaneous activity and we consider that the differences in contractile ability lies within or between the myometrial cells.

Electrical activity i.e. changes of membrane potential and firing of action potentials underlies the rise of Ca within myometrial cells, which is the trigger for contraction. The Ca signals are transmitted between cells via gap junctions and co-ordination of these changes is required for labour contractions [35], [36]. Our findings of reduced amplitude and duration of Ca transients in postdates myometrium relates directly to the reduction of contractility found. Although beyond the scope of this study, this finding suggests that one or more of the following may be reduced: incomplete expression or activation of L-type Ca channels or the co-ordination and spread of action potentials and their signals. Our data with oxytocin and high K^+^ would be consistent with reduced L-type Ca channel activity. Thus both stimuli will increase L-type Ca entry and co-ordinate activity throughout the preparation. Both increased the strength of contractions in postdates myometrium but were unable to raise activity to the same level as found in term samples. This is also the case when our data is divided into labouring and non-labouring samples. In active labour, all the changes in contraction associated protein and other changes necessary for labour have occurred. The myometrium of postdates women however still produced less force compared with term. This could also be explained if L-type Ca channels were reduced. We did find that in samples of labouring myometrium, unlike non-labouring, the duration of the contractions, as well as area under the curve, was significantly reduced. This suggests that the duration of the action potentials and hence Ca entry, may be different in postdates myometrium. Recordings of membrane currents and potential would be required to address this more directly. As contraction is reduced in both labouring and non-labouring samples our data also suggest that whatever mechanism led to this, it is not overcome by the hormonal and other changes leading to labour and remains expressed in the myometrial cells, and apparent in this laboratory study. Of note, we find that there is no difference in the ability of postdates myometrium to achieve spontaneous contractile activity *in vitro* when compared to term myometrium. This is also consistent with the suggestion that contractile activity is reduced but not blocked, in postdate myometrium. *In vivo* additional factors, such as endocrine differences originating from the fetus or placenta, may also contribute to the depression of contractility in women with prolonged pregnancies. As mentioned earlier, there are few data addressing the etiology of prolonged pregnancy. Some of the factors identified, i.e. male fetus, ethnicity, fetal abnormality are hard to link with mechanisms reducing uterine force. Others such as BMI or altered endocrine environment are easier, as for example both may influence excitation contraction coupling and hence Ca and force [29], [37], [38].

Before relating our data to previous clinical findings, it is necessary to note the limitations of our study. For this laboratory study we were able to recruit a large number of women (93). As shown in the demographic tables the groups were well matched in terms of maternal characteristics, but there were some differences in the labouring group, notably the clinically anticipated increased number of nulliparous women and increased need for augmentation. As we showed, our findings of decreased contractility in postdates myometrium remained when analysis was confined to all nulliiparous women, indicating that this cannot account for the differences. There were also more postdates labouring samples from women having CS for fetal distress. However, as indicated in our recent work, in term samples from women having CS for fetal distress, myometrial contractility is higher than that in samples from any other group of clinical reason for CS [39]. Thus our findings of decreased contractility in postdates myometrium, despite a high number of inclusions of CS for diagnosis of fetal distress is, if anything, underestimated in the labouring group. The use of therapeutic oxytocin was greater for the postdates women. However we show that reduced contractility remains even with oxytocin-stimulated contractions, making this unlikely to have influenced our data. Furthermore, as administration of oxytocin therapeutically may have diminished myometrial oxytocin receptors or caused them to desensitize [40], we also compared responses to oxytocin after exclusion of those women noted as having labour augmentation with oxytocin. We found that force of contraction and mean integral force under oxytocin were still reduced in postdates myometrium compared to term. Our findings are also supported by the non-labouring group, in which the myometrium may be expected to be in a more homogenous state. It would also be interesting to investigate further, the oxytocin sensitivity by studying the effects of different doses in future studies. Dating of the pregnancy will rarely be without any error, but in our unit with the use of routine first trimester ultrasound dating scans, we consider this error to be far too small to account for our differences, which as shown in Figure 1 were continuous throughout gestation. This finding of the changes in contractility being a continuum with gestation is also consistent with current views on the increased perinatal and maternal risks of prolonged pregnancy i.e. they do not start as soon as a pregnancy reaches 41^+3^, but can be identified earlier e.g. week 40 or 41 [41], [42]. To further increase confidence when we divided patients into term and postdates, we excluded women having a pregnancy extending beyond 41 weeks but no greater than 41 and 2 days, and women with pregnancies before 39 weeks gestation.

Our *in vitro* findings clearly show reduced Ca signalling and contractility in myometrium from women who have prolonged pregnancies. These data are consistent with several clinical and indirect observations on these patients. Reduced uterine activity was found in early *in vivo* tocodynamometer recordings of amniotic pressure changes [27], [43] and abdominal palpitation assessment of uterine activity [44]. No more recent clinical assessments of uterine activity in postdates pregnancies appear to have been made using these methods. There are also epidemiological studies that support a finding of reduced contractile activity in women with prolonged pregnancies. Thus postdates women have been shown in many studies to have longer labours and increased need for CS or operative vaginal delivery [1], [24], [25], [26], [41]. Another indication of poor myometrial activity in postdates pregnancy is the increased risk of post-partum haemorrhage. This risk for postdates women remains even when confounding factors such as BMI and mode of delivery are controlled for and increased with length of pregnancy prolongation [41]. All are consistent with a reduction in myometrial contractility as the pregnancy progresses beyond 40 weeks. It is not known whether the effect is continuous with preterm labour (where dysfunctional labour is relatively rare) or reversed in preterm births as part of a ‘U’ shaped curve.

In summary, there have been few mechanistic studies of myometrium from women with prolonged pregnancies and hence its etiology is largely unknown. This is despite the well-documented increases in fetal and maternal morbidity associated with prolonged pregnancy and challenges to management of the condition. Our study clearly shows that contractility is reduced in women with postdates pregnancies, and that this remains the case even with oxytocin stimulation and when in labour. These findings are consistent with several clinical maternal and perinatal studies that also suggest poorer myometrial contractility. Further studies are now suggested to explore why contractility is reduced. Our finding of reduced Ca transients, indicate that a problem in excitation contraction coupling may be contributing to the etiology of postdates pregnancy.
